# Partial Decellularized Scaffold Combined with Autologous Nasal Epithelial Cell Sheet for Tracheal Tissue Engineering

**DOI:** 10.3390/ijms221910322

**Published:** 2021-09-25

**Authors:** Luong Huu Dang, Shih-Han Hung, Yuan Tseng, Ly Xuan Quang, Nhi Thao Ngoc Le, Chia-Lang Fang, How Tseng

**Affiliations:** 1 International Ph.D. Program in Medicine, College of Medicine, Taipei Medical University, Taipei 110, Taiwan; luonghuudang167@gmail.com (L.H.D.); seedturtle@gmail.com (S.-H.H.); 2 Department of Otolaryngology, Faculty of Medicine, University of Medicine and Pharmacy at Ho Chi Minh City, Ho Chi Minh City 70000, Vietnam; quang.lx@umc.edu.vn; 3 Department of Otolaryngology, School of Medicine, College of Medicine, Taipei Medical University, Taipei 110, Taiwan; 4 Department of Otolaryngology, Wan Fang Hospital, Taipei Medical University, Taipei 116, Taiwan; 5 Graduate Institute of Medical Sciences, College of Medicine, Taipei Medical University, Taipei 110, Taiwan; m120109010@tmu.edu.tw; 6 Graduate Institute of Biomedical Materials and Tissue Engineering, College of Biomedical Engineering, Taipei Medical University, Taipei 110, Taiwan; lethaongocnhi@gmail.com; 7 Department of Pathology, Wan Fang Hospital, Taipei Medical University, Taipei 116, Taiwan; ccllfang@tmu.edu.tw; 8 Department of Pathology, School of Medicine, College of Medicine, Taipei Medical University, Taipei 110, Taiwan; 9 Department of Biochemistry and Molecular Cell Biology, School of Medicine, College of Medicine, Taipei Medical University, Taipei 110, Taiwan

**Keywords:** tissue engineering, tracheal reconstruction, partial decellularized scaffold, nasal epithelial cell sheet

## Abstract

Decellularization has emerged as a potential solution for tracheal replacement. As a fully decellularized graft failed to achieve its purposes, the de-epithelialization partial decellularization protocol appeared to be a promising approach for fabricating scaffolds with preserved mechanical properties and few immune rejection responses after transplantation. Nevertheless, a lack of appropriate concurrent epithelialization treatment can lead to luminal stenosis of the transplant and impede its eventual success. To improve re-epithelialization, autologous nasal epithelial cell sheets generated by our cell sheet engineering platform were utilized in this study under an in vivo rabbit model. The newly created cell sheets have an intact and transplantable appearance, with their specific characteristics of airway epithelial origin being highly expressed upon histological and immunohistochemical analysis. Subsequently, those cell sheets were incorporated with a partially decellularized tracheal graft for autograft transplantation under tracheal partial resection models. The preliminary results two months post operation demonstrated that the transplanted patches appeared to be wholly integrated into the host trachea with adequate healing of the luminal surface, which was confirmed via endoscopic and histologic evaluations. The satisfactory result of this hybrid scaffold protocol could serve as a potential solution for tracheal reconstructions in the future.

## 1. Introduction

To date, a number of studies on tracheal replacement have been published [1,2]. Nevertheless, some obstacles need to be improved before an ideal tracheal substitute becomes a reality. Recently, some researchers have shifted their attention toward biological tracheal scaffolds derived from decellularized tissues [3]. Decellularization via physical, chemical, enzymatic, or combination methods reduces the immunogenicity of the tissue matrix through the removal of all cellular and nuclear components, thus reducing the risk of allograft rejection [4]. However, with the specific character of the trachea in maintaining the airway structure during inspiration, a completed decellularized scaffold seems to not be an appropriate choice for tracheal replacement, due to lack of a mechanical structure after transplantation [5,6]. Instead, alternative approaches to speed up the process of scaffold generation, including the preservation of the original cartilage cells inside the tracheal scaffold, have been proposed [7,8,9]. In our previous study, we introduced a new partial decellularization (PD) protocol that can fabricate a transplantable scaffold with preserved viable cartilage cellular components that remain functional and gradually restore the luminal strength of the tracheal transplant without signs of immune rejection [10]. 

Although some positive results were recorded when applying our previous partial decellularization technique, we observed nonlethal stenosis at the transplanted segment upon long-term observation. Our previous scaffold only solves the cartilage problem, but the re-epithelialization of the luminal surface is wholly based on epithelial migration from the neighboring portions of the host trachea [10]. Unfortunately, evidence obtained from our previous study showed that it took a long time for full epithelial coverage of the luminal surface of the transplanted segment. Without any inhibition of the epithelium layer, there would be a significant fibroblast migration process, leading to eventual scarring and stenosis. Therefore, to prevent restenosis, rapid remodeling of the mucosal layer on the inner surface of the decellularized tracheal graft may be a key strategy, especially at the early postoperative stage after transplantation. 

The idea of this study was inspired by a report by Kanzaki et al. [11], who first introduced the application of tracheal epithelial cell sheets on a prevascularized bioartificial graft to enhance the regeneration of mature pseudostratified columnar epithelium in a rabbit trachea. The reported result suggests that combining cell sheet technology with other biomaterial constructs based on a multifactorial approach could optimize the re-epithelialization of the tracheobronchial airway and establish a potential application of advanced tissue reconstruction clinically. These results encouraged us to consider incorporating the cell sheet technique in our partial decellularization protocol to perhaps overcome the inner lining issues.

**Figure 1:**
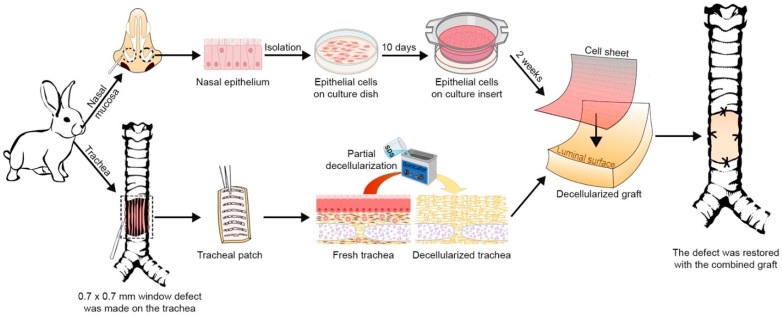
Flow diagram of the research design.

In this study, we generated autologous nasal epithelial cell sheets using our novel cell sheet engineering and then incorporated the cell sheets into transplanted decellularized tracheal scaffolds created by our previous partial decellularization protocol. To our knowledge, this is the first study that has directly applied the cell sheets onto the luminal surface of decellularized tracheal grafts, without any need for a revascularization period, to prevent stenosis formation after transplantation (Figure 1).

## 2. Results

### 2.1. Successful Generation of Autologous Nasal Epithelial Cell Sheets Using Our Novel Cell Sheet Method

**Figure 2:**
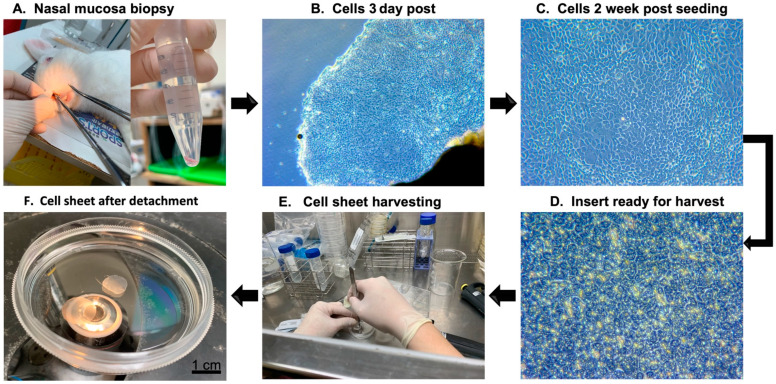
Flow diagram of the fabrication of the transplantable nasal epithelial cell sheets. (**A**): Nasal mucosa was harvested from the nasal cavity; (**B**): epithelial cells started to grow and move outside from the explanted tissue after 3 days of culture (magnification ×40); (**C**): nasal epithelial cells after 2 weeks of culture (magnification ×100); (**D**): cell sheet was ready for harvest after 10 days of seeding on culture insert (magnification ×200); (**E**): the cell sheet was detached from the insert membrane; (**F**): the harvested cell sheet had an intact and transplantable appearance.

First, we successfully created thiol-modified HA-PET inserts, using our novel technique system, which had an inner diameter of 10 mm and a membrane with a 0.4 µm pore size. Then, rabbit nasal epithelial cell sheets were fabricated, using nasal mucosa harvested from the nasal cavity. Nasal epithelial cells were seeded on a 60 mm type I collagen-coated cell culture dish until reaching nearly 80% confluence. Cultured cells were trypsinized, and the obtained cells were seeded on a 1 cm cell culture insert at a density of 5 × 10^5^ cells/insert. After three weeks, nasal mucosal epithelial cell sheets were harvested from the inserts by using reducing agents to cut the functional disulfide bonds between the bottom surface of the attached cell sheets and the inserts. The duration of the detachment process was from 20 to 30 min (Figure 2).

**Figure 3:**
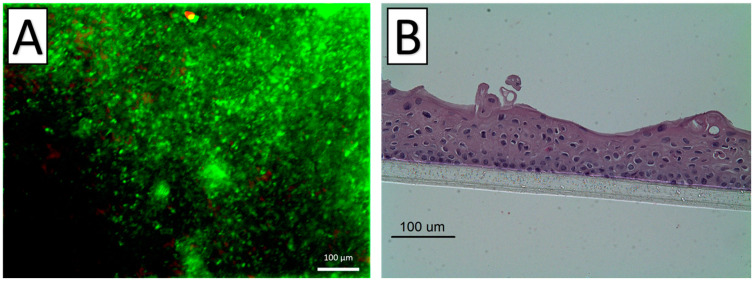
Pretransplantation evaluations of nasal epithelial cell sheets. (**A**): Live–dead staining results showed a high rate of alive cells (green color) in the harvested sheet with a few dead cells (red color). (**B**): The cell sheets consisted of multilayered mucosal epithelial cells with a round-shape appearance under HE staining.

The harvested cell sheet had an intact and transplantable appearance. The viability and functionality of the harvested cell sheet were evaluated immediately after detachment by live–dead staining. The results showed that cell survival was not significantly compromised. Under histological analysis, HE-stained specimens showed that fabricated cell sheets consisted of multilayered mucosal epithelial cells with a round-shaped appearance (Figure 3). 

**Figure 4:**
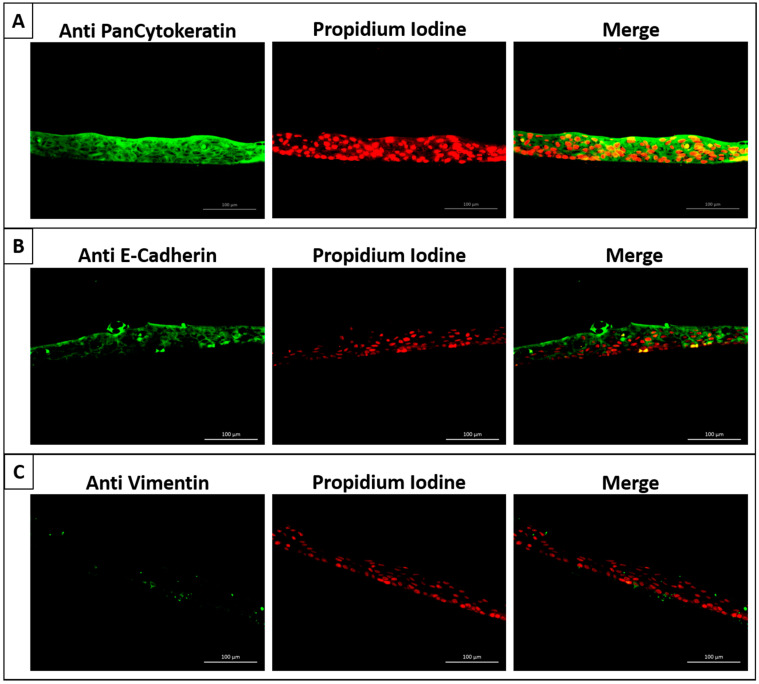
Immunohistochemistry fluorescent staining results of the cell sheets. The cell sheet was stained with each of the following antibodies: (**A**) anti-pan cytokeratin, (**B**) anti-E-cadherin, and (**C**) anti-vimentin. To aid in visualization and facilitating interpretation, all samples were counterstained with propidium iodide, which was used to stain the nucleus. The merged images showed that the nasal epithelial cell sheets expressed highly positive staining to anti-cytokeratin and anti-E-Cadherin antibodies while there was very little expression of anti-vimentin antibodies.

The immunohistochemistry fluorescent staining results revealed that the nasal epithelial cell sheets expressed highly positive staining to anti-cytokeratin and anti-E-Cadherin antibodies, which consistently demonstrated the epithelial character of the sheet. We also noted very little expression of anti-vimentin antibodies in the cell sheet sample (Figure 4).

### 2.2. Results of Surgical Transplantations in Rabbit Groups Using Decellularized Tracheal Grafts with Cell Sheets Applied onto the Luminal Surface

**Figure 5:**
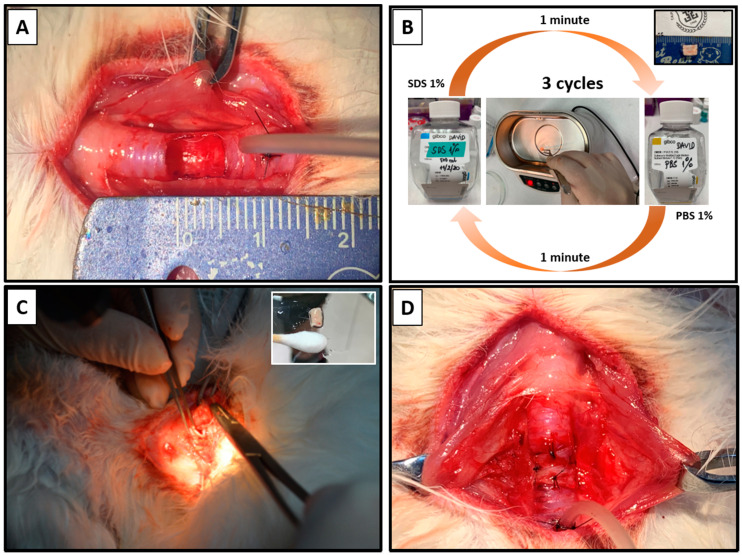
Autograft tracheal patch transplantation process. (**A**): Created defect with a semicylindrical shape measuring approximately 0.7 cm × 0.7 cm on the anterior aspect of the trachea (**B**): The excised part (small photo) underwent the partial decellularization process. (**C**,**D**): The partial decellularized autografts with the cell sheet applied in the luminal surface (small photo) were retransplanted into the original site.

To evaluate the in vivo performance of applying the cell sheet in preventing the stenosis of the decellularized trachea, a partial resection model was used in New Zealand rabbits. By using tracheal autograft transplantation, we could eliminate the role of the immune response in the stenosis formation process and separately evaluate the capacity of the cell sheet to enhance the healing of the luminal surface. The partial decellularized autograft patch was successfully transplanted to the resected portion of the trachea (Figure 5). No animal died during the surgical procedure. 

**Figure 6:**
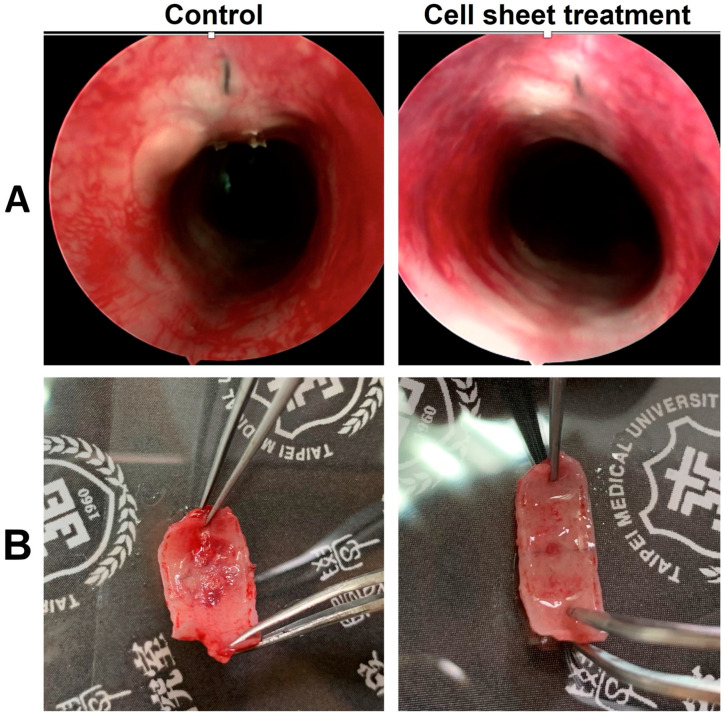
Evaluation of the lumen aspect of the transplanted grafts between two groups. (**A**): Endoscopic view; (**B**): under gross appearance view.

After the operation, all rabbits produced a slight noisy sound when breathing shortly after the operation, but all clinical signs disappeared in the first week, and euthanasia was performed as scheduled. Under endoscopic examination two months post transplantation, the tracheal lumen of the control group showed mild stenosis at the site of transplanted graft eight weeks after transplantation with some outgrowth of granulation tissue on the luminal surface of the graft. The results appeared to be more satisfactory in the cell sheet group in which the epithelium of the tracheal graft appeared thinner and more lustrous. This result was confirmed under postmortem examination of the transplanted graft. Macroscopically, the inner surface of the cell sheet group appeared shinier and smoother than that of the control group (Figure 6).

**Figure 7:**
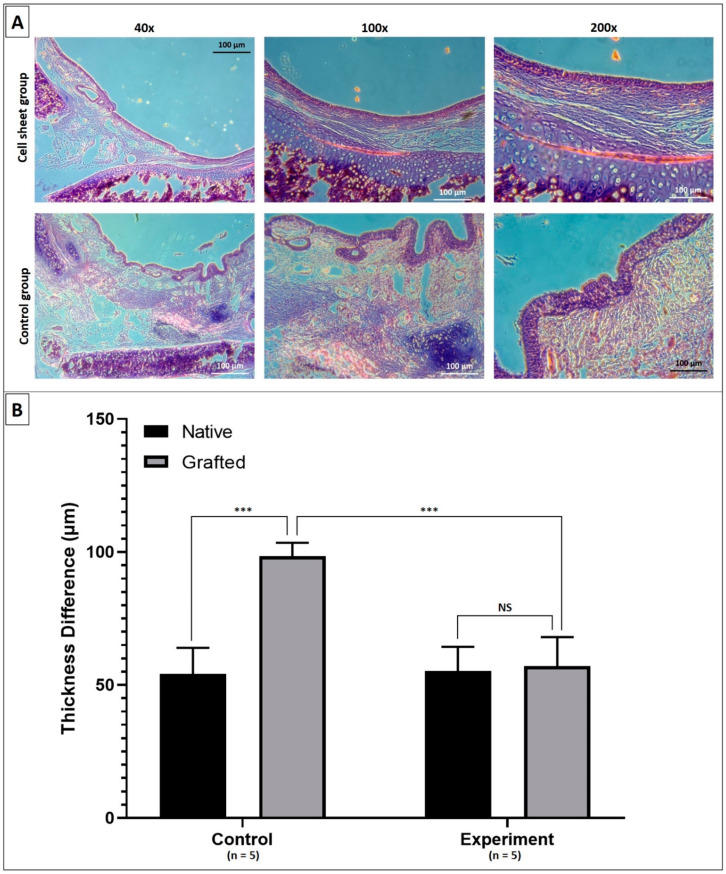
Histological examination of the transplanted grafts at two months post operation. (**A**): HE staining results of the transplanted grafts of the two groups under different magnitude views; (**B**): the graph showed that there was a significant difference in the thickness of the mucosal layer of the transplanted grafts between the two groups. (***) indicates statistical significance with *p* < 0.0001, NS means non-statistical.

Under histological examination, regions at the transplanted graft in both experimental groups demonstrated an intact epithelium in both groups. However, in the control group, we observed dense fibroproliferation with high neovascularization in the subepithelial layer and the hypertrophy of the epithelial layer where the epithelium became thickened. Additionally, this subepithelial fibrosis phenomenon originated from the anastomosis edges that progressed toward the center of the grafts. In contrast, in the group with cell sheet application, subepithelial or intraluminal fibrosis was not observed. That led to a significant difference in the thickness of the mucosal layer of the transplanted graft between the two groups (Figure 7). Upon comparing the results of the transplanted grafts of the two groups at two months post operation, the fibrosis and stenosis problem was improved dramatically with the utilization of the cell sheet technique.

## 3. Discussion

In this study, we demonstrated that through our innovative engineering technology platform, we were able to fabricate an intact and transplantable cell sheet that was cultured from rabbit autologous nasal epithelial cells. This cell sheet was utilized to cover the luminal side of the partial decellularized tracheal graft during transplantation. Using post implantation histology and endoscopy evaluation, we determined that the application of the newly created cell sheet appeared to have the potential to enhance epithelial regeneration and prevent stenosis formation after transplantation of a partial, decellularized tracheal graft. 

To date, several sources of airway epithelial cells have been evaluated for epithelialization of the tracheal luminal surface. The most commonly used is respiratory epithelium harvested from the trachea or nasal mucosa [12]. Unfortunately, tracheal epithelial cells are difficult to harvest from only a small piece of normal tracheal mucosa. The procedure can also result in unnecessary injury to the rabbit trachea. To overcome these problems, we focused on the use of the nasal mucosa as a cell source for preparing the cell sheets since it has been confirmed that the nasal mucosa and lower respiratory mucosa are histologically and characteristically similar [13,14]. At the beginning of this study, a cell sheet composed of highly differentiated respiratory mucosa was created from the nasal epithelial cells (unpublished data). However, we briefly recognized that the mucociliary epithelial cell sheet was unsuitable for a manipulatable graft since its thickness was not strong enough. Jaseok P. Koo et al. [15] reported that squamous epithelial cell sheets cultured in medium lacking retinoid acid demonstrated the advantages of manipulation and retained the ability to differentiate back into fully mucociliary epithelium conditions of a sufficient supply of retinoid acids from the recipient’s serum. Based on this concept, we applied this suggested culture protocol to our study. Consequently, an intact and transplantable nasal epithelial cell sheet was constructed and successfully harvested, which consistently demonstrated the efficiency of our new inserts.

According to our previous study, our fast partial decellularization method has proven the ability to produce low-immunogenicity tracheal allograft scaffolds with preserved viable cartilage cellular components while the epithelial layers were entirely removed. Nevertheless, the absence of luminal lining treatment during transplantation induced a nonlethal stenosis formation in the lumen of the recipient’s trachea. Therefore, we expected that, with the application of the newly established epithelial cell sheet onto the luminal surface of decellularized grafts during transplantation, the healing of the inner lining layer would perform differently than in our previous study. In this study, 10 tracheal patch rabbit models were used and equally divided into two groups. For each model, the anterior part of the trachea was excised and underwent a partial decellularization process before being retransplanted into the original site. At the same time, for retransplantation, one group received the application of epithelial cell sheets onto the inner surface of the decellularized graft. Using two groups of similar patch models, with the only difference being the cell sheet application, the difference in luminal healing post transplantation strongly suggests the efficacy of the cell sheet treatment. As expected, the post transplantation endoscopic and histological results demonstrated that the application of the newly created cell sheet appeared to have the potential to enhance epithelial regeneration and prevent stenosis formation after transplantation of partial decellularized tracheal grafts.

Instead of using an allograft model, the autograft was chosen in this study to eliminate any unpredictable immunorejection response triggered in the recipients, due to any residual of foreign substances in the epithelial layer of the donor tracheal graft after the partial decellularization process that could interfere with the outcome analysis. Even though the autograft model was used, since the tracheal patch also underwent a partial decellularization process before being transplanted, an overgrowth of fibroblasts at the junction and the luminal surface of the transplanted graft was significantly in the group without epithelial treatment, as in our previous study. This result, once again, consolidated the theoretical role of the epithelium in the prevention of stenosis formation. In contrast, there was very little subepithelial or intraluminal fibrosis in the group with cell sheet application, which was confirmed by the significant difference in the thickness of the submucosal layers compared to that of the untreated group. Upon comparing the results of the transplanted grafts of the two groups at two months post operation, the fibrosis and stenosis problem were significantly improved with the utilization of the cell sheet technique.

The most critical issue that we were concerned about before conducting this cell sheet application was whether the cell sheet could survive when transplanted on a graft that had undergone a decellularization process, which completely removes all tissues and blood vessels, which are essential factors for promoting epithelial growth [16]. As reported in many studies that have utilized epithelial cell sheets for surface coverage, the sheets were successfully placed on highly vascularized tissue beds or at least on the structure that had already undergone a period of heterotopic prevascularization [11,17]. To our knowledge, this is the first study that directly introduced the epithelial cell sheet onto the surface of a decellularized graft, without any need for a revascularization period. This could be due to the following reasons: (1) The small graft size facilitated the revascularization from the adjacent tissue. (2) The sheet lay on a native-origin surface of the partial decellularized graft with the remaining ECM compound that could support integration and regeneration. (3) The cell sheet was stably fixed on the graft surface by four supporting sutures during transplantation, enhancing the connection between the grafted sheet and the surrounding healthy tissue, thereby increasing the chance for cell survival. (4) In the autograft model, angiogenesis may be more accessible.

While the application of the epithelial cell sheet presented in this study seems to be very potent for enhancing epithelial regeneration and preventing stenosis formation, there are still some problems that need to be further addressed. In this experimental method, it was still difficult to investigate whether the pseudostratified ciliated epithelial layer covering the transplanted region was the remaining transplanted epithelial cells. It is possible that the transplanted sheet only acts as a bridge for facilitating epithelial migration from the edge of the surrounding host tissue, reducing fibrosis formation. Therefore, labeling the cells in the transplanted sheet with biocompatible indicators, such as fluorescent markers, might help solve this [18]. 

The limitations of this study should also be noted. It is challenging to thoroughly examine the immune rejection response in an autograft model. The question remains of whether these cell sheets also possess benefits in the allograft model, where any residual of donor tissue could immediately elicit an unpredictable reaction of the recipient’s immune system. Therefore, it is necessary to confirm this hypothesis in another study, using the allograft model. Additionally, the application of epithelial cell sheets onto the luminal surface of the whole segment seemed infeasible for this study, due to the lack of appropriate types of equipment for more accessible application onto the lumen. In the future, it is necessary to find a solution in the application of epithelial cell sheets onto the luminal surface of the whole segment in actual clinical practice [19].

## 4. Materials and Methods

### 4.1. Fabrication of Nasal Epithelial Cell Sheets and Pretransplantation Evaluations

#### 4.1.1. Fabrication of Nasal Epithelial Cell Sheets 

##### Preparation of Our Cell Culture Inserts

**Figure 8:**
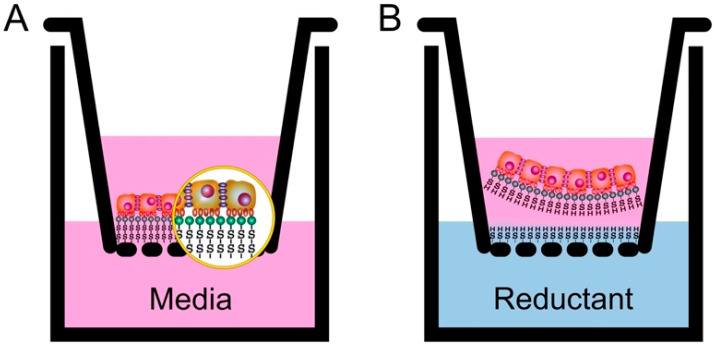
Illustration of the culture inserts and cell sheet detachment used for this study. (**A**): Cells were seeded in the culture insert until they reached the targeted thickness, which could largely vary according to the organ where the cell sheet was applied; (**B**): at the time of detachment, the culture medium inside the well was replaced by the reductant solution, which was used to cut the functional disulfide bonds between the bottom surface of the attached cell sheets and the inserts.

According to our previous publication, a surface chemical modification of porous polyethylene terephthalate (PET) membranes was carried out [20]. First, 0.125 M hyaluronic acid (Kewpie, Japan) was dissolved in 0.25 M boric acid buffer at pH 8.8 and stirred at 4 °C (solution 1). Next, an equivalent EDC/NHS/cystamine (ACROS Organics, Geel, Belgium.) was dissolved in 0.25 M boric acid buffer, pH 6.0 (solution 2), and preactivated for 15 min. After pouring solution 1 into solution 2 and continuing to react for 2 h, the final product of solution 3 was prepared. Second, a 12-well culture insert with a porous PET membrane (pore size: 430 nm, pore density: 5.63 × 10^6^/cm^2^, thickness: 12.5 μm) (ANT Technology, Taipei, Taiwan) was preactivated by carbon dioxide low-pressure plasma (PDC-002-HP, Harrick Plasma, New York, NY, USA), and the pressure was kept at 500 mTorr for 45 min within carbon dioxide atmosphere. The power of the plasma was controlled at 45 W. The PET culture insert was immersed in 0.25 M EDC/NHS in the 0.25 M boric acid buffer at pH 6.0 and 4 °C for 2 h with subsequent mixing with solution 3 of equal volume. Continuous shaking was performed for 4 h. Finally, after completion of the surficial chemical modification, the disulfide bond containing the HA-modified porous HA-PET culture insert was gently washed with water to remove unreacted residues and then air-dried overnight. These prepared culture inserts were sterilized with ethylene oxide gas for further culture use (Figure 8).

##### Culture of Rabbit Nasal Epithelial Cell Sheets

After anesthesia, nasal mucosa was harvested from the nasal cavity using Blakesley forceps and stored in phosphate-buffered saline (PBS) containing 1% penicillin-streptomycin (Thermo Fisher Scientific, Waltham, MA, USA) for 30 min. The harvested tissue was put on a 60-mm, type I collagen-coated cell culture dish (Jet Biofil, Guangzhou, China) in keratinocyte growth medium (KCM) containing 10% fetal bovine serum, hydrocortisone (0.5 mg/mL), insulin (5.0 mg/mL), transferrin (10 mg/mL), triiodothyronine (6.5 ng/mL), epidermal growth factor (0.5 ng/mL), cholera toxin (1 nM), penicillin G sodium (100 U/mL), and streptomycin sulfate (100 mg/mL) [21]. When it reached nearly 80% confluence, the cultured cells were trypsinized, and the obtained cells were seeded on a 1 cm^2^ cell culture insert at a density of 5 × 10^5^ cells/insert. 

After two weeks of subculture in KCM, the fabricated nasal mucosal epithelial cell sheets were harvested from the inserts by adding 3 ml of reductant agent solution, the mixture of 0.13925 g L-Cysteine in 0.5 mL NaOH 1N and 29.5 mL PBS, to the well outside the insert while keeping the medium inside the insert unchanged.

#### 4.1.2. Pretransplantation Evaluations of the Nasal Epithelial Cell Sheets

##### Vital Staining Assay

After completely detaching from the insert, the harvested cell sheets were analyzed for viability, using a vital staining assay. Briefly, the cell sheets were washed three times with PBS and then incubated with a mixture of 60 mM propidium iodide and 10 mM fluorescein diacetate in PBS in the dark for 10 min. Next, the stained cell sheets were washed three times with PBS to remove any residual dye before being observation under a fluorescent microscope.

##### Histological and Immunohistochemical Analyses

For cross-sectional observations, harvested nasal epithelial cell sheets were fixed with 10% neutral buffered formalin and routinely processed into 5 µm-thick paraffin-embedded sections. Hematoxylin and eosin (HE) staining was performed by conventional methods. For immunohistochemistry, deparaffinized sections were washed with PBS, and antigen retrieval was achieved with 10 mM citrate buffer pH 6.0 in a vegetable steamer. The samples were then treated with each of the following antibodies: mouse monoclonal anti-pancytokeratin (1:20 dilution, AE1/AE3, Abcam, Cambridge, UK), mouse monoclonal anti-E-cadherin (1:100 dilution, Abcam, Cambridge, UK) and mouse monoclonal anti-vimentin (1:100 dilution, Merck, Darmstadt, Germany), according to the manufacturer’s suggested protocol. Alexa Fluor 488 goat anti-mouse IgG (Abcam) was used as a secondary antibody, and propidium iodide was used for counterstaining.

### 4.2. Autograft Tracheal Patch Transplantation 

#### 4.2.1. Partial Decellularization of the Rabbit Tracheal Patch 

In this study, we applied our modified partial decellularization method [10]. Each tissue sample will be subjected to the ultrasonic decellularization process in both types of buffers, respectively, with 1% SDS under sonication for 1 min and then 1% PBS under sonication for 1 min as one cycle and repeated three times (3 cycles). The power of the sonication (Elma S 60 H) was set to 180 W. Then, all of the tracheal grafts were stored in an antibiotic solution.

#### 4.2.2. Surgical Transplantation of Autologous Nasal Epithelial Cell Sheets in Autograft Tracheal Patch Transplantation

A total of 10 9-month-old male New Zealand white rabbits acquired from BioLASCO Company (Taipei, Taiwan) with body weights ranging from 3 kg to 3.5 kg were subjects in this study. All animal showed normal breathing before experiments, and were equally divided into two groups.

A partial resection model was used to evaluate the in vivo cell sheet efficacy for preventing stenosis of the decellularized trachea. The cell sheets fabricated from the autologous nasal epithelial cells were prepared in advance for the experimental group as described above. In the operation, after exposure of the trachea, the ventral portion of the trachea was cut into a semicylindrical shape measuring approximately 0.7 cm × 0.7 cm. The excised part of the trachea underwent the partial decellularization process, while the rabbit was maintained under general anesthesia. After applying the cell sheet onto the luminal surface of the decellularized graft, the combined patch was immediately retransplanted into the original defects (*n* = 5). 

Another five rabbits receiving the same operation with a patch of autologous decellularized trachea transplantations without cell sheet application served as the control group. 

After transplantation, the rabbits were orally administered with antibiotics (enrofloxacin 10 mg/kg/day), analgesics (acetaminophen 100 mg/kg/day), and mucolytic medications (acetylcysteine 100 mg/kg/day) for two weeks. During this time, all were strictly observed for any sign of progressively severe dyspnea or any reached humane endpoint.

### 4.3. Endoscopic and Histological Analyses after Transplantation

Endoscopic observation of the trachea was performed on the animals at two months post transplantation by using an Olympus rigid endoscope system (OD = 3 mm; Olympus, Tokyo, Japan). Subsequently, euthanasia was performed by using CO_2_ gas after inducing general anesthesia with Zoletil 100 intramuscularly. Then, the host tracheal structures were immediately harvested with the transplanted segment for gross evaluation and histological examination. The explanted samples were fixed for 24 h in a 10% neutral buffered formalin solution in PBS (pH 7.4) at room temperature, washed in distilled water, dehydrated in graded alcohol, embedded in paraffin, and sectioned at 5 µm. Adjacent sections were stained with H&E (Sigma-Aldrich, St. Louis, MO, USA) and then observed under a microscope.

### 4.4. Statistical Analysis

The thickness of the mucosal layer of the transplanted graft in each image was measured by using ImageJ software (National Institute of Health, New York, NY, USA). Statistical analysis was carried out using Prism 5 (GraphPad, La Jolla, CA, USA). Differences in the thickness of the mucosal layer of the grafted patch with and without cell sheet application were analyzed. Data were expressed as mean ± standard deviation (SD). For the t-test, statistical significance was defined as *p* < 0.05 (*), *p* < 0.001 (**), and *p* < 0.0001(***).

## 5. Conclusions

Although challenges remain that must be overcome before clinical trials can be conducted further in the future, nasal epithelial cell sheet use seems to have good potential for enhancing epithelial regeneration and prevent stenosis formation on the lumen of a decellularized trachea. This merits further investigation.

## 6. Patents

Tseng How has patent #US9,546,349 B2 licensed to Taipei Medical University.
